# Effectiveness of first-line anticancer treatment may predict treatment response in further lines in stage III/IV patients with non-small cell lung cancer

**DOI:** 10.1007/s00432-023-05431-5

**Published:** 2023-09-28

**Authors:** Monika Bratova, Jana Skrickova, Magda Matusikova, Karolina Hrabcova, Libor Havel, Leona Koubkova, Michal Hrnciarik, Jana Krejci, Ondrej Fischer, Martin Svaton, Kristian Brat

**Affiliations:** 1 Department of Respiratory Diseases, University Hospital Brno, Jihlavska Street 20, 625 00 Brno, EU Czech Republic; 2 Faculty of Medicine, Masaryk University, Brno, Czech Republic; 3 Institute of Biostatistics and Analyses, Ltd., Brno, Czech Republic; 4 Department of Respiratory Medicine, Thomayer Hospital, Prague, Czech Republic; 5 Department of Pneumology, University Hospital Motol, Prague, Czech Republic; 6 2nd Faculty of Medicine, Charles University, Prague, Czech Republic; 7 Department of Pneumology, University Hospital Hradec Kralove, Hradec Kralove, Czech Republic; 8 Faculty of Medicine, Charles University in Prague, Hradec Kralove, Czech Republic; 9 Department of Pneumology and Thoracic Surgery, Bulovka Hospital, Prague, Czech Republic; 10 Department of Respiratory Medicine, University Hospital Olomouc, Olomouc, Czech Republic; 11 Faculty of Medicine, Palacky University, Olomouc, Czech Republic; 12 Department of Pneumology, University Hospital Pilsen, Pilsen, Czech Republic; 13 Faculty of Medicine, Charles University in Prague, Pilsen, Czech Republic; 14 International Clinical Research Center, St. Anne‘s University Hospital, Brno, Czech Republic

**Keywords:** Non-small cell lung cancer, Anticancer treatment, Effectiveness, Overall survival, Progression-free survival

## Abstract

**Purpose:**

The aim of our study was to evaluate if therapeutic success in the first-line of anticancer treatments in patients with NSCLC may predict treatment success in the following lines.

**Methods:**

We analyzed the data of patients with NSCLC stage III/IV from the TULUNG registry separately for chemotherapy, TKIs, ALK inhibitors, and immunotherapy in the first line during the years 2011–2019. „Succesful treatment “ was defined as PFS ≥ 6 months, a „good responder “ was a patient with ˃50% of „successful treatment “ lines. Treatment responses were analyzed separately for each drug group. Descriptive statistics, Fisher exact test, Pearson Chi-Squared test, log-rank test, and univariate/multivariate logistic regression models were used.

**Results:**

The first-line TKI therapy was successful in 66.2%, while good responders accounted for 50.7% of the cohort and their rates were similar for all types of TKIs. First-line platinum-based chemotherapy was successful in 43.1% and 48.6% for combinations with pemetrexed and bevacizumab, respectively. Good responders accounted for 29.5% and 25.9%, respectively. In the group of ALK inhibitors, we observed treatment success in 52.3% of cases, while alectinib showed the highest effectiveness (up to 70%). Good responders constituted 50% of the group. In the first-line immunotherapy group, survival benefit was observed in 52.3%, and good responders constituted 52.3% of the cohort.

**Conclusion:**

We concluded that the treatment success in first-line therapies in patients with NSCLC may predict survival benefits in the subsequent lines, particularly in EGFR- or ALK-positive disease and immunotherapy-treated patients.

**Supplementary Information:**

The online version contains supplementary material available at 10.1007/s00432-023-05431-5.

## Introduction

By the 2020s, lung cancer remains one of the most serious cancer diseases, claiming millions of lives worldwide. Despite notable progress in understanding lung cancer pathobiology, further therapeutic improvements are desired. Novel targeted treatments emerged during the last decade and the most apparent progress in treatment outcomes has been achieved in non-squamous non-small cell lung cancer (NSCLC). Importantly, the introduction of novel therapies resulted also in increased opportunities for treatment sequencing in multiple treatment lines.

In daily practice, the real-life effectiveness of various treatments may differ from the efficacy reported from clinical trials (Brat et al. 2020; Cramer-van der Welle et al. 2018), e.g., overall survival (OS) and progression-free survival (PFS) rates for pemetrexed, bevacizumab and tyrosine kinase inhibitors (TKIs) were higher than pooled OS and PFS from clinical trials in our previous study (Brat et al. 2020). Favorable treatment outcomes may be related to the existence of „good responders “—patients benefitting from most types of anticancer therapy. Understanding several new molecular pathways resulted in an increased opportunity for treatment response prediction; however, the relationships in sequenced multilinear treatment are more complex and poorly understood. Some patients with NSCLC respond favorably to multiple treatments and can be generally considered „good responders “. To date, we lack specific biomarkers of good treatment response and favorable outcomes within the use of multiple treatment lines.

The effectiveness of first-line anticancer therapy could be one of such aspects as demonstrated in a study by *Bonotto* et al. (Bonotto et al. 2015). In this study, the authors demonstrated that a 6-month benefit with the first line of anticancer treatment was associated with benefits in the second and further lines in patients with breast cancer (Bonotto et al. 2015). Among the modern-era treatments of NSCLC, pemetrexed is the oldest and most explored regime. A relation between response to the previous treatment line and subsequent treatment success with pemetrexed therapy has been described also in patients with NSCLC (Sun et al. 2010). Similarly, sensitivity to pemetrexed predicted therapeutic benefits from further treatment lines (Park et al. 2019).

In this study, we focused on various classes of first-line anticancer treatments in patients with NSCLC, including platinum-based chemotherapy, tyrosine kinase inhibitors (TKIs), anaplastic lymphoma kinase (ALK) inhibitors, and immunotherapy (IO). Our aim was to evaluate if therapeutic success with certain types of first-line anticancer treatments may predict treatment success in the following lines. We hypothesized that treatment success within first-line therapies may predict the probability of treatment success with subsequent lines.

## Methods

### Subjects

The source of the large-scale data was the TULUNG Registry (a joint registry of the Czech Pneumological Society, the Czech Society for Oncology, and the Institute of Biostatistics and Analyses, Ltd.), a prospective multicenter database of patients with advanced-stage NSCLC treated by modern-era treatments including antifolates, biological agents and/or immunotherapy.

Briefly, the TULUNG registry encompasses prospective data of NSCLC patients from 11 tertiary- or university-type healthcare centers in the Czech Republic. Written informed consent was signed by each patient sharing his/her anonymized data with the scientific community. Patient participation in the study was voluntary. The following data were collected: demography (age, sex, weight, height, body mass index, performance status, race), smoking history, main comorbidities, lung cancer histology, disease stage at time of diagnosis (using the 7^th^ TNM classification) (Mirsadraee et al. 2012), molecular genetic profile (mainly mutation status of EGFR gene), the use of anticancer treatments (including dosage, adverse events and main reasons of treatment discontinuation), radiotherapy or lung surgery, and survival data (overall survival, progression-free survival). The data were collected continuously, and updated regularly on at least twice a year basis.

### Statistics

For this study, we analyzed data of patients with an anticancer treatment initiated between July 1st, 2011 (the foundation of the TULUNG Registry) and December 31st, 2019. The data were analyzed separately for each group of first-line anticancer treatment (chemotherapy, TKIs, ALK inhibitors, and immunotherapy).

Treatment responses were analyzed using the RECIST 1.1 criteria (Eisenhauer et al. 2009). Overall survival (OS) was defined as the time from anticancer treatment initiation to (all-cause) death. Progression-free survival (PFS) was defined as the time from anticancer treatment initiation to the date of first documented disease progression or all-cause death. OS and PFS were estimated using the Kaplan–Meier method and all estimates at various time points included a 95% confidence interval (CI).

„Succesful treatment “ was defined as reaching progression-free survival (PFS) of ≥ 6 months. A „good responder “ was defined as a patient with ˃50% of „successful treatment “ lines of all recorded. Accordingly, a „poor responder “ was defined as a patient with less than 50% of „successful treatment “ lines of all. Treatment responses for each particular drug were analyzed separately and also on a group basis for TKIs, chemotherapy, ALK inhibitors, and IO.

The basic characteristics of the cohort were described by descriptive statistics. Continuous variables were described by means and 95% CI and by medians with minimum and maximum values. Categorical variables were presented as absolute and relative frequencies. Relative frequencies were calculated based on the number of patients in the treatment subgroups. Comparisons between groups were made by the Fisher exact test and Pearson Chi-Squared test.

Differences in PFS were tested by the Log-rank test. Univariate and multivariate logistic regression models were constructed for good and bad responders.

Statistical analyses were performed using IBM SPSS, Statistics (version 25.0), and R software (version 3.5.1).

### Ethics

The study has been approved by the institutional ethics committees of all centers participating in the TULUNG registry (University Hospital Brno, University Hospital Pilsen, University Hospital Olomouc, University Hospital Hradec Kralove, University Hospital Motol (Prague), University Hospital Prague-Bulovka, Thomayer Hospital (Prague), University Hospital Ostrava, Hospital Jihlava, Masaryk Memorial Cancer Institute (Brno) and VFN (Prague)). This particular study was approved by the Ethics Committee of the University Hospital Hradec Kralove on May 11th, 2018, reference number: 201805 I134R.

## Results

We analyzed data from a total of 2317 patients with advanced-stage NSCLC treated in the first line of their anticancer therapy by afatinib (180 patients), gefitinib (340 patients), erlotinib (60 patients), platinum-based chemotherapy plus pemetrexed (1204 patients), platinum-based chemotherapy plus bevacizumab (451 patients), alectinib (24 patients), crizotinib (14 patients), or pembrolizumab (44 patients).

### Tyrosine kinase inhibitors

Five hundred and eighty patients were treated by TKIs during the study period, including 66.2% women with a mean age of 65.9 years and a mean BMI of 26.2 kg/m^2^. Eastern Cooperative Oncology Group performance status (ECOG PS) was mostly 0–1, stage IV patients represented the majority of the cohort. Adverse events (AEs) were reported in 29.7% of cases (Table 1).

**Table 1:** Demography and information of disease

	TKI´s (*N* = 580)	Pemetrexed/ Bevacizumab (*N* = 1655)	ALK inhibitors (*N* = 38)	Pembrolizumab (*N* = 44)
Gender (%)				
Man	196 (33.8%)	974 (58.9%)	12 (31.6%)	26 (59.1%)
Woman	384 (66.2%)	681 (41.1%)	26 (68.4%)	18 (40.9%)
Age (years)				
Mean (95% CI)	65.9 (65.1–66.8)	63.1 (62.6–63.5)	61.2 (58.0–64.3)	66.4(63.8–69.1)
Smoking status (%)				
Non-smoker	322 (55.5%)	332 (20.1%)	26 (68.4%)	7 (15.9%)
Former smoker	151 (26.0%)	578 (34.9%)	5 (13.2%)	11 (25.0%)
Current smoker	107 (18.4%)	745 (45.0%)	7 (18.4%)	26 (59.1%)
BMI	*N* = *525*	*N* = *1411*	*N* = *37*	*N* = *43*
Mean (95% CI)	26.2 (25.8–26.6)	26.3 (26.1–26.6)	26.1 (24.4–27.9)	27.1 (25.8–28.4)
ECOG PS (%)				
0	154 (26.6%)	454 (27.4%)	14 (36.8%)	15 (34.1%)
1	392 (67.6%)	1159 (70.0%)	22 (57.9%)	27 (61.4%)
2	30 (5.2%)	41 (2.5%)	2 (5.3%)	2 (4.5%)
3	4 (0.7%)	1 (0.1%)	0 (0%)	0 (0%)
Race (%)	*N* = *577*	*N* = *1649*		
Caucasian	564 (97.6%)	1641 (99.5%)	37 (97.4%)	44 (100%)
Black	0 (0%)	1 (0.1%)	1 (2.6%)	0 (0%)
Asian	14 (2.4%)	7 (0.4%)	0 (0%)	0 (0%)
Stage of disease (%)				
III	49 (8.4%)	204 (12.3%)	2 (5.3%)	0 (0%)
IV	531 (91.6%)	1451 (87.7%)	36 (94.7%)	44 (100%)
Adverse events (%)				
Yes	172 (29.7%)	223 (13.5%)	5 (13.2%)	10 (22.7%)
No	408 (70.3%)	1432 (86.5%)	31 (81.6%)	34 (77.3%)

*TKIs* Thyrosine kinase inhibitors, *ALK* Anaplastic Lymphoma Kinase, *BMI* Body Mass Index, *PS* Performance Status

The first-line therapy was successful in 66.2% while the effectiveness of further lines gradually decreased with increasing treatment lines. No differences in the effectiveness of individual TKIs were observed across the lines (Table 2). Good responders for the TKIs group accounted for 50.7% of the cohort and their rates were similar for all TKIs. The probability of a ≥ 6-month PFS after a TKI did not differ for any of the TKIs. The ten most frequent treatment patterns/sequences are presented in Table 3. Of the EGFR mutations, the deletion in exon 19 dominated (53.1% of all patients), while the L858R mutation in exon 21 was less frequent with 26%, and other mutations (the T790M and other rare mutations) constituted a total of 26.4% (Table 4). The proportion of EGFR mutations was similar/balanced in all TKI subgroups (Table 4). In the multivariate analysis, age and Exon 19 deletion were the only factors independently associated with being a good responder while former smoking was associated with a poor responder (Supplementary Table 1).

**Table 2:** Patients with a 6-month survival benefit in TKI group/all patients (%)

	All (*N* = 580)	Afatinib (*N* = 180)	Gefitinib (*N* = 340)	Erlotinib (*N* = 60)	*p-value*
All	294 (50.7%)	96 (53.3%)	170 (50.0%)	28 (46.7%)	0.620
1st line	384/580 (66.2%)	124/180 (68.9%)	224/340 (65.9%)	36/60 (60.0%)	0.441
2nd line	86/207 (41.5%)	28/70 (40.0%)	49/120 (40.8%)	9/17 (52.9%)	0.628
3rd line	22/69 (31.9%)	8/27 (29.6%)	14/39 (35.9%)	0/3 (0.0%)	0.610
4th line	6/16 (37.5%)	1/6 (16.7%)	5/10 (50.0%)	0/0 (0.0%)	
5th line	0/2 (0.0%)	0/0 (0.0%)	0/2 (0.0%)	0/0 (0.0%)	
6th line	0/1 (0.0%)	0/1 (0.0%)	0/0 (0.0%)	0/0 (0.0%)

**Table 3:** Patients with/without a survival 6-month benefit in TKI group (%)

Lines of treatment 1st 2nd 3rd 4th 5th 6th	All (*N* = 580)	Afatinib (*N* = 180)	Gefitinib (*N* = 340)	Erlotinib (*N* = 60)
+	222 (38.3%)	69 (38.3%)	131 (38.5%)	22 (36.7%)
-	145 (25.0%)	39 (21.7%)	86 (25.3%)	20 (33.3.%)
+ -	65 (11.2%)	19 (10.6%)	40 (11.8%)	6 (10.0%)
+ +	41 (7.1%)	14 (7.8%)	21 (6.2%)	6 (10.0%)
- -	19 (3.3%)	8 (4.4%)	11 (3.2%)	0 (0.0%)
+ -	17 (2.9%)	5 (2.8%)	10 (2.9%)	2 (3.3%)
- +	14 (2.4%)	1 (0.6%)	10 (2.9%)	3 (5.0%)
+ + -	11 (1.9%)	6 (3.3%)	5 (1.5%)	0 (0.0%)
+ + +	7 (1.2%)	3 (1.7%)	4 (1.2%)	0 (0.0%)
- - -	5 (0.9%)	3 (1.7%)	2 (0.6%)	0 (0.0%)

**Table 4:** Proportion of EGFR mutations in the TKI group

	All (*N* = 580)	Afatinib (*N* = 180)	Gefitinib (*N* = 340)	Erlotinib (*N* = 60)
Deletion in exon 19	308 (53.1%)	103 (57.2%)	178 (52.4%)	27 (45.0%)
L8R8R exon 21	151 (26.0%)	37 (20.6%)	100 (29.4%)	14 (23.3%)
Other	153 (26.4%)	54 (30.0%)	86 (25.3%)	13 (21.7%)

The subgroup of patients treated by osimertinib in the second line included a significantly higher proportion of patients with a 6-month benefit (60.5%) (Supplementary Table 2), as well as good responders (55%) (Supplementary Table 3) when compared to patients on platinum-based chemotherapy (41.9% and 34.5%) or other treatment (mostly chemo-monotherapy) (34.6% and 28%; *p* = 0.021 and <0.001, respectively).

### Chemotherapy with pemetrexed or bevacizumab

Chemotherapeutic regimens with pemetrexed or bevacizumab were used in 1655 patients, of these 58.9% were men with a mean age of 63.1 years and a mean BMI of 26.3 kg/m^2^. Most patients were diagnosed with stage IV disease and had an initial ECOG PS 0-1. Treatment AEs were reported in 13.5% of patients (Table 1).

The rate of patients with a ≥ 6-month PFS with first-line pemetrexed- or bevacizumab-based regimens was 43.1% and 48.6%, respectively. In the pemetrexed subgroup, we observed 29.5% of good responders, while only 25.9% in the bevacizumab-based regimen subgroup. There was no difference between the two subgroups regarding survival benefit in any of the subsequent lines, i.e., the effectiveness was similar for both regimens (Table 5). The ten most frequent treatment sequences/patterns are presented in Table 6. In the multivariate analysis, age was associated with a good responder, while higher ECOG PS at treatment initiation with a poor responder (Supplementary Table 4).

**Table 5:** Patients with a 6-month survival benefit in chemotherapy group/all patients (%)

	All (N = 1655)	Pemetrexed (N = 1204)	Bevacizumab (N = 451)	p-value
All	472 (28.5%)	355 (29.5%)	117 (25.9%)	0.155
1st line	738/1655 (44.6%)	519/1204 (43.1%)	219/451 (48.6%)	0.052
2nd line	159/675 (23.6%)	82/392 (20.9%)	77/283 (27.2%)	0.066
3rd line	40/229 (17.5%)	21/91 (23.1%)	19/138 (13.3%)	0.077
4th line	8/24 (33.3%)	6/13 (46.2%)	2/11 (18.2%)	0.211
5th line	1/10 (10.0%)	1/4 (25.0%)	0/6 (0.0%)	0.400
6th line	0/1 (0.0%)	0/0 (0.0%)	0/1 (0.0%)	–

**Table 6:** Patients with/without 6-month benefit in chemotherapy group (%)

Lines of treatment 1st 2nd 3rd 4th 5th 6th	All (*N* = 1655)	Pemetrexed (*N* = 1204)	Bevacizumab (*N* = 451)
-	549 (33.2%)	450 (37.4%)	99 (22.0%)
+	346 (20.9%)	282 (23.4)	64 (14.2%)
- -	237 (14.3%)	173 (14.4%)	64 (14.2%)
+ -	180 (10.9%)	127 (10.5%)	53 (11.8%)
+ +	65 (3.9%)	47 (3.9%)	18 (4.0%)
+ - -	45 (2.7%)	2 (0.2%)	43 (9.5%)
- +	37 (2.2%)	25 (2.1%)	12 (2.7%)
+ -	33 (2.0%)	30 (2.5%)	3 (0.7%)
- - -	31 (1.9%)	3 (0.2%)	28 (6.2%)
- -	27 (1.6%)	26 (2.2%)	1 (0.2%)

For the second line of treatment, there was no significant difference in the proportion of patients with a 6-month benefit among groups with immunotherapy, platinum-based chemotherapy, and other treatments (mostly chemo-monotherapy, e.g., docetaxel, gemcitabine, and vinorelbine) (Supplementary Table 5). On the other hand, in the first-line chemotherapy group, we observed a higher proportion of good responders in the second line with IO and platinum-based chemotherapy compared to the subgroup of chemo-monotherapy (24.7% and 33.7% vs 15.5%, respectively, *p* < 0.001) (Supplementary Table 6).

### ALK inhibitors

In the studied period, alectinib or crizotinib was used in the first line of anticancer treatment in thirty-six ALK-positive patients. In this group, men constituted 31.6% of the cohort, with a mean age of 61.2 years and a mean BMI of 26.1 kg/m^2^. Unlike in other treatment groups, 68.4% were non-smokers. Initial stage IV disease was diagnosed in 94.7% of patients (Table 1).

Alectinib was significantly more effective than crizotinib in the first-line therapy of ALK-positive patients (Table 7). Good responders constituted 50% of the entire group; however, the result was largely driven by alectinib (66.7% of good responders compared to 21.4% for crizotinib, *p* = 0.007). The six most common treatment sequences/patterns are presented in Table 8. Age was the only independent factor of a poor responder with ALK inhibitors (Supplementary Table 7).

**Table 7:** Patients with a 6-month survival benefit in ALK inhibitor group/all patients (%)

	All (*N* = 38)	Alectinib (*N* = 24)	Crizotinib (*N* = 14)	p-value
All	19 (50.0%)	16 (66.7%)	3 (21.4%)	**0.007**
1st line	21/38 (55.3%)	17/24 (70.8%)	4/14 (28.6%)	**0.018**
2nd line	1/8 (12.5%)	0/2 (0.0%)	1/6 (16.7%)	>0.999
3rd line	0/1 (0.0%)	0/0 (0.0%)	0/1 (0.0%)	*-*

Statistically significant changes are indicated in bold

**Table 8:** Patients with/without a 6-month survival benefit in ALK inhibitors group (%)

Lines of treatment 1st 2nd 3rd	All (*N* = 38)	Alectinib (*N* = 24)	Crizotinib (*N* = 14)
+	18 (47.4%)	16 (66.7%)	2 (14.3%)
-	12 (31.6%)	6 (25.0%)	6 (42.9%)
- -	4 (10.5%)	1 (4.2%)	3 (21.4%)
+ -	2 (5.3%)	1 (4.2%)	1 (7.1%)
+ +	1 (2.6%)	0 (0.0%)	1 (7.1%)
- - -	1 (2.6%)	0 (0.0%)	1 (7.1%)

### Immunotherapy (IO)

From 2011 to 2019, pembrolizumab was the only first-line check-point inhibitor available for use in the Czech Republic for patients with a stage IV NSCLC with PD-L1 expression of ˃50%. Pembrolizumab monotherapy was initiated in 44 patients during the study period. In this group, men accounted for 59.1% of the cohort, the mean age was 66.4 years, and the mean BMI was 27.1 kg/m^2^. All patients were diagnosed with stage IV. AEs of IO were observed in 22.7% of patients (Table 1).

First-line survival benefit (≥ 6-month PFS) was observed in 52.3% of patients. Treatment benefit from the second line was the same and good responders constituted 52.3% of the cohort (Table 9). The treatment sequences/patterns are presented in Table 10. In the multivariate analysis, a statistically insignificant trend towards a good responder was observed in former/current smokers and patients experiencing AEs (Supplementary Table 8).

**Table 9:** Patients with 6-month benefit in immunotherapy group/all patients (%)

	All (*N* = 44)
All	23 (52.3%)
1st line	23/44 (52.3%)
2nd line	3/6 (50.0%)
3rd line	0/2 (0.0%)

**Table 10:** Patients with/without 6-month benefit in immunotherapy group (%)

Lines of treatment 1^st^ 2^nd^ 3^rd^	All (*N* = 44)
+	21 (47.7%)
-	16 (36.4%)
- -	3 (6.8%)
+ + -	1 (2.3%)
+ +	1 (2.3%)
- +	1 (2.3%)
- -	1 (2.3%)

An inter-group comparison of good responders across all treatment groups showed a significantly higher (almost doubled) rate of good responders in groups of biological therapy and IO when compared to the chemotherapy-based groups (Supplementary Table 9).

## Discussion

To our best knowledge, this was the first study attempting to assess survival benefits in its complexity during all lines of patients´ anticancer therapies in patients with NSCLC. A similar study has been performed by Bonotto et al. in 2015 (Bonotto et al. 2015). The authors of this study described the most frequent sequences/patterns of anticancer therapies in a cohort of 472 patients with breast cancer and assessed if treatment response of PFS ≥ 6 months within the first line of treatment resulted in a higher probability of survival benefit with further treatment lines. As an extension of this study design, we attempted to assess treatment responses for all lines in groups of patients with NSCLC treated by different first-line therapies.

The main finding of our study was that patients treated by certain groups of anticancer therapies (EGFR inhibitors, ALK inhibitors, IO) had an increased probability of experiencing a ≥ 6-month survival benefit not only in their first line of anticancer treatment but also in subsequent treatment lines. This observation was most apparent in patients treated with alectinib (ALK inhibitor). On the contrary, patients treated with chemotherapy-based regimens experienced the lowest rate of survival benefit in the first and also in the subsequent lines of their anticancer therapies.

The observed favorable outcomes with EGFR inhibitors, ALK inhibitors, and IO have been demonstrated previously in a number of studies.

For both EGFR- and ALK-positive patients, our data showed that the rate of good responders was significantly higher than in the chemotherapy-based group. We speculate that these tumor types may be composed of heterogenic (but dominantly EGFR- or ALK-mutant) cancer cells that are prone to significant treatment response with targeted therapies. Residual cancer tissue may harbor a broader number and spectrum of oncogenic driver mutations with an overall weaker potential for rapid progression of the disease after targeted treatment.

To date, the most frequently used biological therapy in patients with NSCLC is the group of EGFR inhibitors (TKIs). Several predictive markers exist, indicating a potential therapeutic benefit from TKIs. These include Exon 19 deletion as some subtypes of this mutation may have an important clinical impact (Huang et al. 2022) or the plasma level of circulating tumor DNA (ctDNA) as lower ctDNA during the treatment course is associated with better survival (Kallergi et al. 2022; Provencio et al. 2021).

Several TKIs (erlotinib, gefitinib, afatinib, dacomitinib, and osimertinib) can be used in various treatment lines, and also in a sequence. The optimal sequencing strategy appeared to be the combination of afatinib in the first line, followed by second-line osimertinib in a case of a proven T790M mutation (Hirsh et al. 2020; Kim et al. 2023), until the results of the FLAURA study have been published, shifting osimertinib to the first-line therapy of EGFR positive NSCLC (Lorenzi et al. 2022). Exact recommendations on treatment after first- or second-line osimertinib failure were not formulated. Usually, a switch to chemotherapy or chemo-immunotherapy is preferred (Reck et al. 2019). In our study, we found larger numbers of treatment benefits and good responders among patients treated by osimertinib in the second line which is in accordance with current knowledge (Hirsh et al. 2020; Kim et al. 2023).

According to recent studies, PD-L1 expression of ≤ 1% (Lasvergnas et al. 2023) and concomitant genetic mutations AXIN2, P2CG, or RAD51C may predict a poor response to a first-line TKI therapy (Wen et al. 2023). On the other hand, post-treatment TKIs-resistant NSCLC tumors have recently been considered potentially targetable by MET-, HER2-, and HER3-directed therapies (Johnson et al. 2022).

For ALK-positive NSCLC, several ALK inhibitors can be used in a sequence (alectinib, ceritinib, crizotinib, brigatinib, lorlatinib) (Kauffman et al. 2021). Alectinib, brigatinib and lorlatinib, ALK inhibitors with high intracerebral penetration, are recommended as first-line drugs. Lorlatinib is preferred in the second line of treatment post-failure after a second-generation ALK inhibitor (ESMO guidelines 2023; Hochmair et al. 2020). On the other hand, a recent study by Takeyasu et al. found similar effectivity of second-line lorlatinib and pemetrexed-based chemotherapy after failure of alectinib (Takayasu et al. 2022).

After crizotinib failure, a variety of other ALK drugs can be used (ESMO guidelines 2023). Survival of patients with NSCLC harboring ALK mutation and receiving an ALK inhibitor seems to be independent of the fusion variants of ALK translocation (Tabbó et al. 2022). In contrast, other authors discuss worse effectiveness in patients harboring variant 3 of the ALK translocation (Lin et al. 2018). Further studies are needed to uncover the mechanisms of resistance in ALK-mutated NSCLC disease.

Another group of highly effective anticancer treatment for NSCLC is immunotherapy. It gains a still larger therapeutic scope not only in the treatment of NSCLC but also in small-cell lung cancer (SCLC) or malignant pleural mesothelioma. To date, expression of PD-L1 remains the key marker of treatment response to IO (Herbst et al. 2014; Kim et al. 2018). However, new markers of treatment response emerged, including ECOG PS, presence of brain metastases, molecular genetic factors, circulating regulatory T cells, and immune-related adverse events (irAEs). Circulating regulatory T cells are associated with better response during IO and may help to differentiate between disease pseudoprogression and hyperprogression (Kang et al. 2022). As to the molecular genetic factors, patients with a concurrent KRAS- or BRAF-positive disease experienced longer PFS when compared to those with an EGFR- or ALK-positive profile (Bodor et al. 2022). By contrast, ECOG PS ≥ 2 or the presence of brain metastases were associated with early progression of the disease (Dumenil et al. 2018). Patients developing irAEs are known to benefit more significantly compared to those without irAEs; the more irAEs the patient experiences, the higher the probability of a survival benefit from IO (Sonehara et al. 2021).

Similarly to our observation, better survival outcomes after first-line IO have also been reported in a retrospective multicenter study by Bersanelli et al. (Bersanelli et al. 2020). In this study, the authors observed improved survival and ORR after chemotherapy in patients pre-treated with IO, concluding that IO may increase the tumor´s sensitivity to the (subsequent) chemotherapy (Bersanelli et al. 2020). Our data seem to support this hypothesis.

In contrast, low TMB has been found a predictor of responsiveness to first-line chemotherapy (PFS 9.77 vs 6.33 months, HR = 0.523; *p* = 0.009) (Song et al. 2022) while other authors report the tumor mutation index model (based on total/sensitive TMB from blood and mutation score) as a potential predictor of response to chemotherapy or immunotherapy in the subsequent treatment lines (Lu et al. 2022). Nastase et al. identified a group of genes associated with a good response to platinum chemotherapy in IIIA stage NSCLC (Nastase et al. 2022). Genetic background of treatment response to adjuvant chemotherapy has also been observed in patients with low-stage NSCLC (Van Laar 2012).

In summary, the differences in treatment responses to various classes of first-line anticancer therapies observed in our study likely point out the complexity of mechanisms of tumor pathogenesis in NSCLC. Different tumor subpopulations may coexist in each individual patient, and this may evolve also during the course of their anticancer therapy. Further research is needed to understand the molecular and genetic mechanisms and the broader clinical context beyond our observation.

### Limitations

There are several limitations of this study. First, the study is retrospective, however, based on large-scale data that provide relevant information regarding real-life experience with anticancer therapy of NSCLC in the Czech Republic. Second, the data are not fully representative of the entire Czech Republic as it comes from 11 tertiary- or university-type healthcare centers and do not fully represent the situation in the whole Czech healthcare system. Third, we did not differentiate between various types of chemotherapeutic regimens in detail as this would make the interpretation of the results almost impossible. Fourth, the ALK and IO subgroups are relatively small as these therapies were introduced just at the end of the study period. Last, we considered only limited treatment options for the advanced stages of NSCLC. In the study period, chemo/immunotherapy was not used commonly in the first-line anticancer strategy compared to the current clinical practice. Despite these limitations, we consider the presented data strong, generalizable, and representative of the treatment strategies in the Czech Republic.

## Conclusion

We concluded that the effectiveness of first-line therapies in patients with NSCLC may predict survival benefit in the subsequent lines, particularly in EGFR-, ALK-, or PD-L1-positive disease treated by targeted therapies. By contrast, chemotherapy-based regimens were associated with the highest rate of poor response in all lines of anticancer treatment. Further studies are needed to uncover and better understand the underlying mechanisms of increased treatment responsiveness in certain subgroups of patients with NSCLC.

## Supplementary Information

Below is the link to the electronic supplementary material.

Supplementary file1 (DOCX 33 KB)

## Data Availability

The datasets generated and/or analyzed during the study are available from the corresponding author on a reasonable request.
