# Supplementary material for: Effectiveness of first-line anticancer treatment may predict treatment response in further lines in stage III/IV patients with non-small cell lung cancer
Source: J Cancer Res Clin Oncol. 2023 Sep 28;149(19):17123–31. doi: 10.1007/s00432-023-05431-5 (PMC10657273; doi:10.1007/s00432-023-05431-5)
Supplement: Supplementary file 1 — Supplementary file1 (DOCX 33 KB) [file 432_2023_5431_MOESM1_ESM.docx]

**Effectiveness of first-line anticancer treatment may predict treatment response in further lines in stage III/IV patients with non-small cell lung cancer.**

[Journal of Cancer Research and Clinical Oncology](https://www.springer.com/journal/432)

Monika Bratova^1,2^, Jana Skrickova^1,2^, Magda Matusikova^3^, Karolina Hrabcova^3^,

Milos Pesek^4,5^, Libor Havel^6^, Leona Koubkova^7,8^, Michal Hrnciarik^9,10^, Jana Krejci^11^, Ondrej Fischer^12,13^, Martin Svaton^4,5^ and Kristian Brat^1,2,14^

*Correspondence to:*

Kristian Brat, MD, PhD, assoc.prof.

Department of Respiratory Diseases

University Hospital Brno and Faculty of Medicine, Masaryk University

Jihlavska street 20, 625 00 Brno, Czech Republic, EU

Email: brat.kristian@fnbrno.cz

Tel: +420 532 232 556

***Supplementary Table 1:*** Logistic regression for good/poor responders in TKI group

|  | **Univariate analysis** | | **Multivariate analysis** | |
| --- | --- | --- | --- | --- |
|  | **OR (95% CI)** | **P-value** | **OR (95% CI)** | **P-value** |
| **Gender (%)**  Man  Woman | Reference category  1.18 (0.84-1.67) | *-*  *0.348* | Reference category  0.91 (0.62-1.33) | *-*  *0.638* |
| **Age (years)** | 1.02 (1.00-1.03) | ***0.014*** | 1.02 (1.01-1.04) | ***0.004*** |
| **Smoking status (%)**  Non-smoker  Former smoker  Current smoker | Reference category  0.52 (0.35-0.77)  0.61 (0.39-0.95) | *-*  ***0.001***  ***0.028*** | Reference category  0.57 (0.37-0.86)  0.74 (0.46-1.18) | *-*  ***0.009***  *0.205* |
| **ECOG PS (%)**  0  1  2-3 | Reference category  0.68 (0.47-0.99)  0.65 (0.31-1-37) | *-*  ***0.044***  *0.256* | Reference category  0.72 (0.49-1.07)  0.66 (0.30-1.45) | *-*  *0.101*  *0.303* |
| **Stage of disease (%)**  III  IV | Reference category  0.63 (0.34-1.13) | *-*  *0.124* | Reference category  0.71 (0.38-1.33) | *-*  *0.292* |
| **Comorbidity**  No  Yes | Reference category  0.60 (0.31-1.14) | *-*  *0.124* | Reference category  0.55 (0.27-1.06) | *-*  *0.076* |
| **Adverse events (%)**  No  Yes | Reference category  1.43 (1.00-2.05) | *-*  ***0.050*** | Reference category  1.37 (0.94-1.99) | *-*  *0.101* |
| **Exon 19 deletion**  No  Yes | Reference category  1.69 (1.22-2.35) | *-*  ***0.002*** | Reference category  1.72 (1.22-2-43) | *-*  ***0.002*** |

***Supplementary Table 2:*** Patients treated by TKI in the 1^st^ line – 6-month benefit/all patients according to the type of 2^nd^ line treatment

|  | **Osimertinib**  **in the 2^nd^ line**  **(N=38)** | **Chemotherapy**  **in the 2^nd^ line**  **(N=62)** | **Other tretament**  **in the 2^nd^ line**  **(N=107)** | **P-value** |
| --- | --- | --- | --- | --- |
| **2^nd^ line 6- month treatment benefit** | 23 (60.5%) | 26 (41.9%) | 37 (34.6%) | ***0.021*** |

***Supplementary Table 3:*** Good and poor responders in TKI groups by 2^nd^ treatment line (%)

|  | **Osimertinib**  **in the 2^nd^ line**  **(N=38)** | **Chemotherapy**  **in the 2^nd^ line**  **(N=70)** | **Other tretament**  **in the 2^nd^ line**  **(N=107)** | **P-value** |
| --- | --- | --- | --- | --- |
| **Good responders** | 21 (55%) | 24 (34.5%) | 30 (28%) | ***<0.001*** |

***Supplementary Table 4:*** Logistic regression for good/poor responders in chemotherapy group

|  | **Univariate analysis** | | **Multivariate analysis** | |
| --- | --- | --- | --- | --- |
|  | **OR (95% CI)** | **P-value** | **OR (95% CI)** | **P-value** |
| **Gender (%)**  Man  Woman | Reference category  1.09 (0.87-1.35) | *-*  *0.453* | Reference category  1.10 (0.88-1.38) | *-*  *0.638* |
| **Age (years)** | 1.02 (1.01-1.03) | ***0.001*** | 1.02 (1.01-1.04) | ***<0.001*** |
| **Smoking status (%)**  Non-smoker  Former smoker  Current smoker | Reference category  1.00 (0.74-1.34)  0.87 (0.65-1.16) | *-*  *0.984*  *0.337* | Reference category  1.00 (0.74-1.37)  0.95 (0.70-1.28) | *-*  *0.985*  *0.724* |
| **ECOG PS (%)**  0  1  2-3 | Reference category  0.64 (0.51-0.81)  0.43 (0.18-0.90) | *-*  ***<0.001***  ***0.036*** | Reference category  0.61 (0.48-0.78)  0.38 (0.16-0.81) | *-*  ***<0.001***  ***0.018*** |
| **Stage of disease (%)**  III  IV | Reference category  0.83 (0.61-1.15) | *-*  *0.259* | Reference category  0.86 (0.62-1.18) | *-*  *0.341* |
| **Comorbidity**  No  Yes | Reference category  1.24 (0.81-1.86) | *-*  *0.312* | Reference category  1.22 (0.79-1.84) | *-*  *0.358* |
| **Adverse events (%)**  No  Yes | Reference category  1.04 (0.76-1.41) | *-*  *0.823* | Reference category  1.05 (0.76-1.43) | *-*  *0.763* |

***Supplementary Table 5:*** Patients treated by chemotherapy in the 1^st^ line – 6- month benefit/all patients according to the type of 2^nd^ line treatment

|  | **Immunotherapy**  **in the 2^nd^ line**  **(N=73)** | **Chemotherapy**  **in the 2^nd^ line**  **(N=98)** | **Other tretament**  **in the 2^nd^ line**  **(N=592)** | **P-value** |
| --- | --- | --- | --- | --- |
| **2^nd^ line** | 23/73 (31.5%) | 3/10 (30%) | 133/592 (22.5%) | *0.188* |

***Supplementary Table 6:*** Good responders in the chemotherapy groups after the 2^nd^ treatment line (%)

|  | **Immunotherapy**  **in the 2^nd^ line**  **(N=73)** | **Chemotherapy**  **in the 2^nd^ line**  **(N=98)** | **Other tretament**  **in the 2^nd^ line**  **(N=592)** | **P-value** |
| --- | --- | --- | --- | --- |
| **Good responders** | 18 (24%) | 33 (33.7%) | 92 (15.5%) | ***<0.001*** |

***Supplementary 7:*** Logistic regression for good/poor responders in ALK inhibitor group

|  | **Univariate analysis** | | **Multivariate analysis** | |
| --- | --- | --- | --- | --- |
|  | **OR (95% CI)** | **P-value** | **OR (95% CI)** | **P-value** |
| **Gender (%)**  Man  Woman | Reference category  1.00 (0.25-4.00) | *-*  *>0.999* | Reference category  0.44 (0.02-5.11) | *-*  *0.547* |
| **Age (years)** | 0.92 (0.84-0.99) | ***0.029*** | 0.87 (0.74-0.98) | ***0.041*** |
| **Smoking status (%)**  Non-smoker  Former smoker  Current smoker | Reference category  0.42 (0.05-2.94)  0.10 (0.01-0.73) | *-*  *0.380*  ***0.050*** | Reference category  1.10 (0.03-26.23)  0.04 (0.00-0.68) | *-*  *0.950*  *0.061* |
| **ECOG PS (%)**  0  1-2 | Reference category  0.40 (0.10-1.51) | *-*  *0.183* | Reference category  0.69 (0.12-3.86) | *-*  *0.669* |
| **Stage of disease (%)**  III  IV | Reference category  1.00 (0.04-26.61) | *-*  *>0.999* | Reference category  0.22 (0.00-9.42) | *-*  *0.403* |
| **Comorbidity**  No  Yes | Reference category  0.70 (0.12-3.71) | *-*  *0.676* | Reference category  1.19 (0.06-24.07) | *-*  *0.904* |
| **Adverse events (%)**  No  Yes | Reference category  0.63 (0.08-4.26) | *-*  *0.633* | Reference category  0.11 (0.00-2.68) | *-*  *0.185* |

***Supplementary 8:*** Logistic regression for good/poor responders in immunotherapy group

|  | **Univariate analysis** | | **Multivariate analysis** | |
| --- | --- | --- | --- | --- |
|  | **OR (95% CI)** | **P-value** | **OR (95% CI)** | **P-value** |
| **Gender (%)**  Man  Woman | Reference category  0.86 (0.25-2.88) | *-*  *0.802* | Reference category  1.09 (0.23-5.26) | *-*  *0.901* |
| **Age (years)** | 1.04 (0.97-1.12) | *0.331* | 1.05 (0.97-1.15) | *0.234* |
| **Smoking status (%)**  Non-smoker  Former smoker  Current smoker | Reference category  6.67 (0.91-69.73)  2.50 (0.45-19.74) | *-*  *0.078*  *0.321* | Reference category  11.35 (0.93-248.00)  4.73 (0.61-56.54) | *-*  *0.080*  *0.166* |
| **ECOG PS (%)**  0  1-2 | Reference category  0.94 (0.26-3.29) | *-*  *0.919* | Reference category  1.38 (0.29-6.93) | *-*  *0.687* |
| **Comorbidity**  No  Yes | Reference category  0.48 (0.14-1.59) | *-*  *0.235* | Reference category  0.30 (0.06-1.29) | *-*  *0.120* |
| **Adverse events (%)**  No  Yes | Reference category  2.63 (0.62-13.81) | *-*  *0.211* | Reference category  4.84 (0.83-38.49) | *-*  *0.099* |

***Supplementary 9:*** Good and poor responders in all groups (%)

|  | **TKI´s**  **(N=180)** | **Chemotherapy**  **(N=60)** | **ALK inhibitors**  **(N=38)** | **Immunotherapy**  **(N=44)** | ***P value*** |
| --- | --- | --- | --- | --- | --- |
| **Good responder** | 294 (50.7%) | 472 (28.5%) | 19 (50.0%) | 23 (52.3%) | ***<0.001*** |
| **Poor responder** | 286 (49.3%) | 1183 (71.5%) | 19 (50.0%) | 21 (47.7%) |  |
